# Esophageal Cancer Metabolite Biomarkers Detected by LC-MS and NMR Methods

**DOI:** 10.1371/journal.pone.0030181

**Published:** 2012-01-23

**Authors:** Jian Zhang, Jeremiah Bowers, Lingyan Liu, Siwei Wei, G. A. Nagana Gowda, Zane Hammoud, Daniel Raftery

**Affiliations:** 1 Department of Chemistry, Purdue University, West Lafayette, Indiana, United States of America; 2 Weldon School of Biomedical Engineering, Purdue University, West Lafayette, Indiana, United States of America; 3 Cardiothoracic Surgery, Henry Ford Hospital, Detroit, Michigan, United States of America; I2MC INSERM UMR U1048, France

## Abstract

**Background:**

Esophageal adenocarcinoma (EAC) is a rarely curable disease and is rapidly rising worldwide in incidence. Barret's esophagus (BE) and high-grade dysplasia (HGD) are considered major risk factors for invasive adenocarcinoma. In the current study, unbiased global metabolic profiling methods were applied to serum samples from patients with EAC, BE and HGD, and healthy individuals, in order to identify metabolite based biomarkers associated with the early stages of EAC with the goal of improving prognostication.

**Methodology/Principal Findings:**

Serum metabolite profiles from patients with EAC (n = 67), BE (n = 3), HGD (n = 9) and healthy volunteers (n = 34) were obtained using high performance liquid chromatography-mass spectrometry (LC-MS) methods. Twelve metabolites differed significantly (*p*<0.05) between EAC patients and healthy controls. A partial least-squares discriminant analysis (PLS-DA) model had good accuracy with the area under the receiver operative characteristic curve (AUROC) of 0.82. However, when the results of LC-MS were combined with 8 metabolites detected by nuclear magnetic resonance (NMR) in a previous study, the combination of NMR and MS detected metabolites provided a much superior performance, with AUROC = 0.95. Further, mean values of 12 of these metabolites varied consistently from healthy controls to the high-risk individuals (BE and HGD patients) and EAC subjects. Altered metabolic pathways including a number of amino acid pathways and energy metabolism were identified based on altered levels of numerous metabolites.

**Conclusions/Significance:**

Metabolic profiles derived from the combination of LC-MS and NMR methods readily distinguish EAC patients and potentially promise important routes to understanding the carcinogenesis and detecting the cancer. Differences in the metabolic profiles between high-risk individuals and the EAC indicate the possibility of identifying the patients at risk much earlier to the development of the cancer.

## Introduction

Esophageal cancer is a deadly disease with an estimated 16,640 new cases and 14,500 deaths in the United States in 2010 [1]. In the year 2000 the corresponding numbers were 12,300 and 12,100, respectively [2], which indicate a significant rise in incidence. Of the two cancer types, esophageal adenocarcinoma (EAC) and squamous cell carcinoma, EAC is more prevalent in the United States. Although the risk factors associated with the EAC are not clearly understood to date, Barrett's esophagus (BE) is considered to be a factor for the carcinogenesis of the esophagus [3]. In addition, high-grade dysplasia (HGD) is considered as an immediate precursor to invasive adenocarcinoma [4]. However, no intervention currently exists that can prevent the progression of BE or HGD to EAC [5]. The traditional methods for diagnosing esophageal cancer, including endoscopy and barium swallow, suffer from poor specificity and sensitivity, which typically result in detection of the disease only at an advanced stage [6], [7], [8], [9]. Alternatively, it is hoped that a certain subset of molecular biomarkers can characterize the stage of the disease and help personalize treatment [10]. At the molecular level, carcinogenesis of esophagus is thought to be a complex process involving multiple genetic abnormalities and environmental factors. Numerous studies report specific alterations in proteins, genes and metabolic pathways in EAC that may be useful to aid in the diagnosis, prognosis and treatment of esophageal cancer [11], [12], [13], [14]. Microarray studies have also focused discovery of new markers based on individual tumor genetic composition [15]. However, reliable markers, especially at an early and potentially curative stage, are still in great demand.

Metabolomics, also commonly known as metabolic profiling and metabonomics, is a fast growing field in systems biology and offers a powerful and promising approach to identify biomarkers associated with cancer and other diseases. Metabolomics focuses on deriving the concentrations and fluxes of low molecular weight metabolites (<∼1 kDa) in biofluids or tissue, which provide detailed information on biological systems and their current status [16], [17]. Mass spectrometry (MS) and nuclear magnetic resonance (NMR) spectroscopy are the two most powerful and commonly used analytical methods for metabolic fingerprinting [17], [18]. The two methods are complementary; while MS is highly sensitive, NMR is highly quantitative and reproducible. Utilization of both MS and NMR methods leads to the routine analysis of over 1000 metabolites.

A growing number of metabolomics studies have been reported for detecting various cancers [19], [20], [21], [22], [23]. However, studies that focus on EAC are still relatively small in number. Recently, two papers reported on the analysis of tissue metabolites using magic-angle spinning (MAS) NMR and gas chromatography (GC) MS each combined with multivariate statistical methods [24], [25]. Both works reported a number of statistically significant distinguishing metabolites. Another ^1^H NMR study investigated human plasma and identified variations in several metabolite concentrations associated with EAC that differed among ethnic groups [26]. Efforts in our laboratory have been focused on the development of metabolomics tools and biomarker candidates to detect early EAC and to identify patients at high risk of developing EAC. We recently reported metabolomics-based investigations of EAC using ^1^H NMR spectroscopy and showed that eight serum metabolites differentiated EAC from healthy controls [27]. We also targeted a number of nucleosides in serum using liquid chromatography-triple quadruple (LC-QqQ) MS and showed very significant variations in a number of normal and methylated nucleosides in EAC [28]. With the goal of enhancing the sensitivity and specificity of the patient classification as well as identifying individuals at risk of developing the cancer, in this study we applied global metabolic profiling approach to the serum samples from EAC, BE and HGD patients, and healthy controls using highly sensitive and resolved time-of-flight mass spectrometry coupled with liquid chromatography (LC-TOF) MS. Metabolic profiles were analyzed separately and in combination with previously derived metabolite markers using NMR methods [27]. We evaluated the combination of NMR and MS data in terms of their performance in classifying EAC patients and healthy controls when compared to the performance of either MS or NMR data alone. The ability of the metabolic profiles to distinguish high-risk individuals (BE and HGD patients) from EAC as well as healthy controls was examined. We also identified a number of metabolites that acted as trending markers, in that their mean levels increased/decreased continuously from healthy controls to high-risk subjects and then EAC patients.

## Materials and Methods

### Chemicals

Deuterium oxide (99.9% D) was purchased from Cambridge Isotope Laboratories, Inc. (Andover, MA). Trimethylsilylpropionic acid-d_4_ sodium salt (TSP), tridecanoic acid, chlorophenylalanine, lactic acid, valine, leucine, isoleucine, methionine, carnitine, tyrosine, tryptophan, myristic acid, margaric acid, linolenic acid, linoleic acid and pyroglutamic acid were purchased from Sigma-Aldrich (analytical grade, St. Louis, MO). 5-hydroxytryptophan was purchased from Alfa-Aesar (analytical grade, Ward Hill, MA). HPLC-grade methanol and acetic acid were purchased from Fisher Scientific (Pittsburgh, PA). Deionized water was obtained from an EASYpure II UV water purification system (Barnstead International, Dubuque, IA).

### Serum sample collection and storage

Fasting blood samples from patients with histologically proven EAC (n = 67), HGD (n = 9) and BE (n = 3) were collected at the Indiana University School of Medicine (Indianapolis, IN). The detailed clinicopathologic characteristics of EAC patients were described in our previous paper [27], and a summary is shown in Table S1. We used 68 EAC, 11 HGD and 5 BE samples in the previous NMR study. However, due to the limited amounts of some samples, we removed 1 EAC, 2 HGD and 2 BE samples for the LC-MS experiments and further analysis in this work; the corresponding NMR data was also excluded from the combined analysis and discussion. Blood samples from healthy volunteers (n = 34) were obtained under fasting conditions. Each blood sample was allowed to clot for 45 min and then centrifuged at 2,000 rpm for 10 min. The serum was collected, aliquoted in a separate vial, frozen, and shipped over dry ice to Purdue University (West Lafayette, IN), where they were stored at −80°C until use. All samples were collected following the protocol approved by Indiana University School of Medicine and Purdue University Institutional Review Boards. All subjects included in the study provided written informed consent according to institutional guidelines.

### Sample preparation and data acquisition

For LC-MS analysis, frozen serum samples were thawed, and the protein was precipitated by mixing 100 µL serum and 200 µL cold methanol. Two internal standards, tridecanoic acid and chlorophenylalanine were also included to monitor the extraction efficiency. The mixture was centrifuged at 13200 rpm for 10 min. The supernatant solution obtained after protein removal was dried under vacuum and the obtained residue was reconstituted in 15 µL methanol/water (1∶1) solution. The resulting solution was again centrifuged at 13200 rpm for 10 min to remove particulate matter, if any, and the supernatant was transferred to an LC vial. Separately, a pooled sample was obtained by mixing together 20 µL from each of 20 human serum samples randomly selected from all the samples, and the metabolites were extracted using the same procedure as above. This pooled sample, referred to as the quality control (QC) matrix sample, was subjected to analysis periodically between every 10 samples. QC sample data also served as technical replicates throughout the data set to assess process reproducibility. LC-MS analysis was performed using an Agilent LC-QTOF system (Agilent Technologies, Santa Clara, CA) consisting of an Agilent 1200 SL liquid chromatography system coupled online with an Agilent 6520 time-of-flight mass spectrometer. A 3 µL aliquot of reconstituted sample was injected onto a 2.1×50 mm Agilent Zorbax Extend-C18 1.8 µm particle column with a 2.1×30 mm Agilent Zorbax SB-C8 3.5 µm particle guard column, which were both heated to 60°C. Serum metabolites were gradient-eluted at 600 µL/min using mobile phase A: 0.2% acetic acid in water and mobile phase B: 0.2% acetic acid in methanol (2% to 98% B in 13 min, 98% B for 6 min). Electrospray ionization (ESI) was used in positive mode. The MS interface capillary was maintained at 325°C, with a sheath gas flow of 9 L/min. The spray voltage for positive ion injection was 4.0 kV. The mass analyzer was scanned over a range of 50–1000 *m*/*z*. Agilent MassHunter Workstation LC-TOF and QTOF Acquisition software (B.02.01) was used for automatic peak detection and mass spectrum deconvolution.

Detailed procedures for sample preparation and NMR experiments were recently published elsewhere [27]. Briefly, frozen serum samples were thawed, and 200 µL was mixed with 350 µL of D_2_O. Resulting solutions were transferred to 5-mm NMR tubes. A 60 µL solution of TSP (0.12 mg/mL) in a sealed capillary was utilized as an internal standard, which acted as the chemical shift reference (δ = 0.00). All ^1^H NMR experiments were carried out at 25°C on a Bruker DRX-500 spectrometer equipped with a triple resonance ^1^H inverse detection probe with triple axis magnetic field gradients. ^1^H NMR spectra were acquired using the standard one-dimensional CPMG (Carr-Purcell-Meiboom-Gill) pulse sequence with water signal presaturation. Each dataset was averaged over 64 transients using 16 K time domain points. The data were Fourier transformed after multiplying by an exponential window function with a line broadening of 1 Hz, and the spectra were phase and baseline corrected using Bruker TopSpin software (version 3.0).

### Data analysis

LC-MS data was processed using Agilent's MassHunter Qualitative Analysis software (version B.03.01) for compound identification. A list of ion intensities for each detected peak was generated using a retention time (RT) index and m/z data as the identifiers for each ion. Agilent MassHunter Workstation Mass Profiler Professional software (version B.02.00) was then used for compound peak alignment. A filter was set to remove any metabolite signals that had missing peaks (ion intensity = 1) in more than 10% of the samples in any group. Peaks from internal standards were also removed. Finally, the Agilent Formula Database (Agilent, 2010) was used for compound identification by matching the accurate mass spectrum to a database of metabolite compounds. Unpaired Student's *t*-test analysis of the data was performed to assess the differences of detected compound intensities among EAC, BE and HGD samples, and healthy controls. Metabolites with low *p*-values (<0.05) were selected as potential biomarker candidates and verified from the mass spectra and RTs of authentic commercial compounds run separately. The fold change (FC) for each metabolite was calculated to determine metabolite's variation between the groups.

NMR spectral regions were binned to 4 K buckets of equal width (1.5 Hz) to minimize errors due to any fluctuations of chemical shifts arising from pH or ion concentration variations. Each spectrum was aligned to the methyl peak of alanine at 1.48 ppm, and normalized using the integrated TSP signal. Spectral regions of 0.3 to 10.0 ppm were used for the analysis after deleting the water and urea signals (4.5 to 6.0 ppm). Univariate analysis was performed by applying the unpaired Student's *t*-test to identify significantly different spectral bins among EAC, BE and HGD patients, and healthy controls. Bins that showed significant differences between various patient/controls groups were then assigned to the corresponding metabolites by comparing chemical shifts and multiplicities of peaks to the literature or online databases [29], [30], [31]. The characteristic spectral regions for each metabolite were integrated, and *p*-values and fold changes between different groups were calculated.

The MS/NMR data of the selected statistically significant metabolites (with *p*<0.05) were imported into Matlab (R2008a, Mathworks, Natick, MA) installed with a PLS toolbox (version 4.1, Eigenvector Research, Inc., Wenatchee, WA) for PLS-DA analyses. The X matrix, consisting of the MS/NMR spectral data, was autoscaled prior to all statistical analyses. Depending on the group, each subject was assigned a “0” (i.e., patient) and “1,” (i.e., healthy control) to serve as the (one-dimensional) Y matrix. Leave-one-out cross validation (CV) was chosen, and the number of latent variables (LVs) was selected according to the minimum root mean square error of CV procedure. Predictions were made visually using a Y-predicted scatter plot with a cut-off value chosen to minimize errors in class membership. The R statistical package (version 2.8.0) was used to generate receiver operating characteristics (ROC) curves, calculate and compare sensitivity, specificity and area under the ROC curve (AUROC).

## Results

The LC-MS spectrum for each serum sample consisted of more than 5000 features of which nearly 1400 peaks were assigned to metabolites using the Agilent database. Peaks from the spectra that were missing in more than 10% of the samples from any group were omitted from further analysis. The use of this filter and the Agilent chemical library resulted in a total of approximately 200 indentified metabolites most common to all the groups. Further, to identify specific metabolites that best correlated with the differences in biological status for the various comparisons, the library-identified metabolites were analyzed using univariate analysis. The results showed that 40 metabolites varied significantly (*p*<0.05) between either EAC and healthy controls, EAC and high-risk patients (BE and HGD patients), or high-risk patients and healthy controls. Thirteen of these metabolites could be verified from the mass spectra and retention times of the authentic commercial compounds. Table S2 shows the list of the verified metabolites from LC-MS along with their formulae, masses and retention times. Similarly, as shown in Table S3, fifteen patient-class differentiating metabolites with low *p*-values (*p*<0.05) obtained by integrating the relevant NMR peaks were confirmed by matching the observed chemical shifts and multiplicities with the previously reported data [29], [30], [31].

The summary of the metabolite biomarker candidates from LC-MS and NMR with their *p*-values and fold changes are shown in Table 1. ANOVA was also performed, with results that closely paralleled those from the *t*-test (Table S4). However, since we were interested in identifying individual markers that distinguished each of the three patient cohorts separately, we used the *t*-test data to identify markers for model building. The sensitivity, specificity and AUROC values from the PLS-DA models of each comparison are listed in Table 2. Comparison of MS and NMR data using the *t*-test, separately, showed no significant differences due to gender, age or cancer stage (*p*>0.05) between EAC and controls (Table S5).

**Table 1:** Differentiating metabolites (*p*-value <0.05) among EAC, high-risk (BE and HGD) and control groups.

Metabolite	Detection	EAC *vs* Control		EAC *vs* High-risk		High risk *vs* Control	
		*p*-value^a^	FC^b^	*p*-value^a^	FC^b^	*p*-value^a^	FC^b^
lactic acid	LC-MS	1.2E-07	1.6	3.4E-02	1.6		
	NMR	2.7E-03	1.3			1.6E-02	1.4
valine	LC-MS	2.9E-07	−1.6	1.0E-02	1.6		
	NMR			3.7E-02	1.2		
leucine/isoleucine^c^	LC-MS	2.7E-07	−1.2	4.2E-02	1.2		
methionine	LC-MS	2.0E-05	−1.6	2.4E-02	-1.6		
carnitine	LC-MS	5.7E-05	1.2				
tyrosine	LC-MS	4.0E-03	−1.1				
	NMR			3.7E-02	1.2		
tryptophan	LC-MS	3.2E-05	−1.2				
5-hydroxytryptophan	LC-MS	2.6E-02	−1.1				
myristic acid	LC-MS	1.2E-03	−1.4	1.8E-02	−1.4		
margaric acid	LC-MS	9.5E-03	1.3				
linolenic acid	LC-MS	1.5E-02	−1.4	4.3E-02	−1.2		
linoleic acid	LC-MS	1.1E-04	−1.5				
pyroglutamic acid	LC-MS			9.2E-06	2.0	1.4E-04	−2.2
glutamine	NMR	3.0E-02	1.1				
β-hydroxybutyrate	NMR	2.3E-05	1.3				
citrate	NMR	3.3E-04	1.3				
unknown 1	NMR	3.0E-05	1.3				
lysine	NMR	9.6E-04	1.1	2.8E-02	1.2		
creatinine	NMR	2.2E-02	1.2				
glucose	NMR	1.5E-04	1.2				
N-acetylated protein	NMR			6.4E-04	1.2	3.7E-02	−1.1
proline	NMR			3.1E-03	−2.7	1.3E-02	2.1
histidine	NMR			7.4E-03	1.3		
alanine	NMR			9.2E-03	1.3		
glutamate	NMR			3.6E-02	1.2		
unknown 2	NMR					1.4E-02	−1.5

a*p*-value determined from Student's *t*-test, only *p*-values <0.05 are displayed;

bFC: fold change between esophageal adenocarcinoma (EAC) and healthy controls. Positive sign indicates a higher level in EAC and a negative value indicates a lower level;

cThe structural isomers of leucine and isoleucine could not be separated with the current LC method.

**Table 2:** Comparison of sensitivity, specificity and AUROC values from different PLS-DA models using differentiating metabolites detected individually by NMR or MS and their combination.

Comparison	Number of candidate markers		Sensitivity	Specificity	AUROC
	MS	NMR			
**EAC ** ***vs*** ** Control**	12	-	77%	86%	0.82
	-	8	82%	88%	0.86
	12	8	91%	91%	0.95
	8^a^	4^a^	89%	90%	0.92
**EAC ** ***vs*** ** High-risk**	7	-	83%	80%	0.87
	-	8	77%	77%	0.72
	7	8	67%	97%	0.82
	8^a^	4^a^	75%	70%	0.78
**High-risk ** ***vs*** ** Control**	1	-	74%	75%	0.76
	-	4	68%	92%	0.80
	1	4	65%	92%	0.80

aTrending markers that progressively change in their levels between EAC, high risk (BE and HGD) and healthy controls (see Figure 3).

### Comparing metabolic profiles between EAC patients and healthy controls

As shown in Table 1, twelve metabolite marker candidates detected by LC-MS differentiated EAC patients and healthy controls, and their identities were confirmed with authentic compounds. Figure S1 shows the box-and-whisker plots for the peak intensities of the 12 differentiating biomarker candidates. As seen in Table 1 and Figure S1, the levels of lactic acid, carnitine and margaric acid were higher, and those of valine, leucine/isoleucine (these structural isomers could not be separated with the current LC method), methionine, tyrosine, tryptophan, 5-hydroxytryptophan, myristic acid, linolenic acid and linoleic acid were lower in EAC patients compared to healthy controls.

The marker candidates from ^1^H NMR analysis have been reported in our previous study [27]. Briefly, a set of 8 metabolites, including β-hydroxybutyrate, lysine, glutamine, citrate, creatinine, lactate, glucose and an unknown molecule were statistically significant (*p*<0.05), and higher levels of each of those metabolites in the EAC specimens were observed (Table 1).

**Figure 1:**
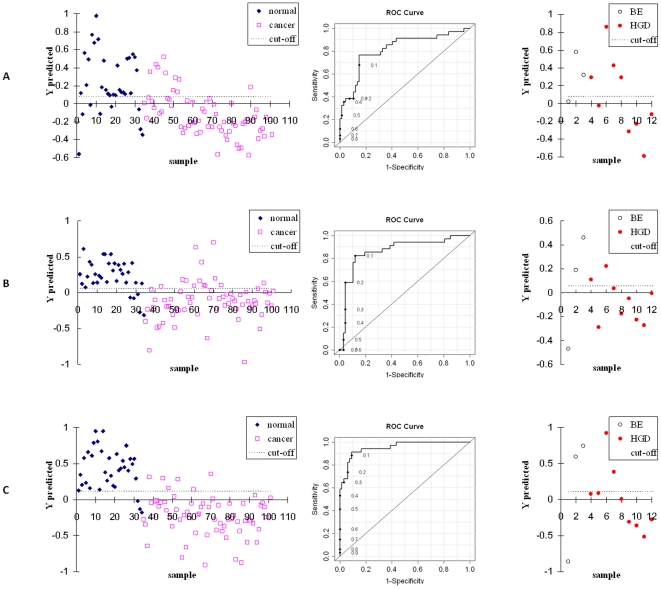
Performance comparison of metabolic profiles between EAC patients and healthy controls. (A) Left, result of the PLS-DA model using 12 metabolite markers from LC-MS analyses; middle, ROC curve using the cross-validated predicted class values (AUROC = 0.82); right, PLS-DA prediction for the BE and HGD samples from the LC-MS model comparing EAC and healthy controls. (B) Same as (A) except using 8 metabolite markers from NMR analyses, (AUROC = 0.86); (C) Same as (A) except using the combination of LC-MS and NMR detected metabolite markers, (AUROC = 0.95).

Figure 1 shows the comparison of performance of 3 metabolic profiles between EAC patients and healthy controls. A PLS-DA model using the twelve LC-MS derived metabolites (and leave-one-out cross valuation) provided 77% sensitivity and 86% specificity with an AUROC of 0.82. Similar analysis using the eight NMR derived metabolites provided 82% sensitivity and 88% specificity with an AUROC of 0.86. However, when the metabolite data were analyzed combining the 12 LC-MS and the 8 NMR detected metabolites, the model provided much superior performance with both sensitivity and specificity of 91%, and an AUROC of 0.95.

To evaluate the BE and HGD samples, the same PLS-DA model was applied, and the result is also shown in Figure 1 (at the right). BE samples gave a mixed result, and no confident conclusion could be made because of the small number of samples. However, most of the HGD samples were predicted as EAC in this case. The PLS-DA model based on NMR detected metabolites, and the model based on combining LC-MS and NMR detected metabolites both showed that 7 out of 9 HGD patients were indicated as being similar to EAC samples.

### Comparing metabolic profiles of EAC and high-risk patients

The data for high-risk patients (BE and HGD patients) were combined for the analysis because of their small sample numbers. Univariate analysis of the data showed that 7 LC-MS and 8 NMR detected metabolites varied significantly between EAC and the high-risk patients, which along with the *p*-values and fold changes are shown in Table 1.

**Figure 2:**
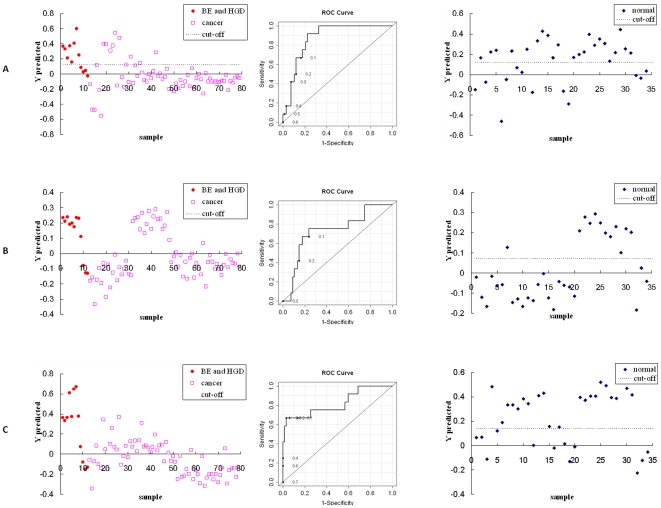
Performance comparison of metabolic profiles between EAC patients and those with high-risk esophageal diseases (BE and HGD). (A) Left, result of the PLS-DA model using 7 metabolite markers from LC-MS analyses; middle, ROC curve using the cross-validated predicted class values (AUROC = 0.87); right, PLS-DA prediction for the healthy controls using the model developed using LC-MS metabolites comparing EAC and high-risk patients. (B) Same as (A) except using the 8 metabolite markers from NMR analyses, (AUROC = 0.72). (C) Same as (A) except using the combination of LC-MS and NMR detected metabolite markers, (AUROC = 0.82).

PLS-DA models were then built using the LC-MS and NMR derived metabolite signals, separately and in combination, to test the classification accuracy for the two patient groups, and the results are shown in Figure 2. The LC-MS derived metabolites provided sensitivity and specificity of 83% and 80%, respectively, with an AUROC of 0.87, and NMR derived metabolites provided both sensitivity and specificity of 77% with an AUROC of 0.72. When the data were analyzed combining the LC-MS and NMR derived metabolites, a sensitivity and specificity of 67% and 97% were obtained, respectively, with an AUROC of 0.82. Here, although the performance of the model from the combined data was slightly better than that from NMR data alone, the model derived from the LC-MS detected metabolites showed the best performance. When testing the controls using the same PLS-DA models derived from the LC-MS detected, NMR detected and combined metabolites, 22, 12 and 22 of 34 controls were above the cut-off value, respectively, and were therefore classified as not being similar to EAC patients.

### Comparing metabolic profiles of healthy controls and high-risk patients

Only one metabolite, pyroglutamic acid, detected by LC-MS, and three NMR detected metabolites, proline, lactic acid and an unknown metabolite, differed significantly (*p*<0.05) between high-risk patients from healthy controls (Table 1). In addition, a peak arising from N-acetylated protein in the NMR spectra showed a significant difference between the two groups. While the levels of pyroglutamic acid, proline and lactic acid were higher in the high-risk group, the others were lower.

The LC-MS and NMR data for the high-risk individuals and healthy controls were compared using PLS-DA analysis (Figure S2). The lone distinguishing metabolite detected by LC-MS, pyroglutamic acid, had a sensitivity and specificity of 74% and 75%, respectively, with an AUROC of 0.76. A PLS-DA model based on the NMR detected metabolites provided a sensitivity and specificity of 68% and 92%, respectively, with an AUROC of 0.80. The combined analysis of the data from the two analytical methods provided results similar to that for NMR alone. However, all the models failed to give a clear prediction of the EAC patients using only the high risk and healthy cohorts, indicating that (not unexpectedly) EAC patient samples are needed to build a successful detection model.

### Trending markers

**Figure 3:**
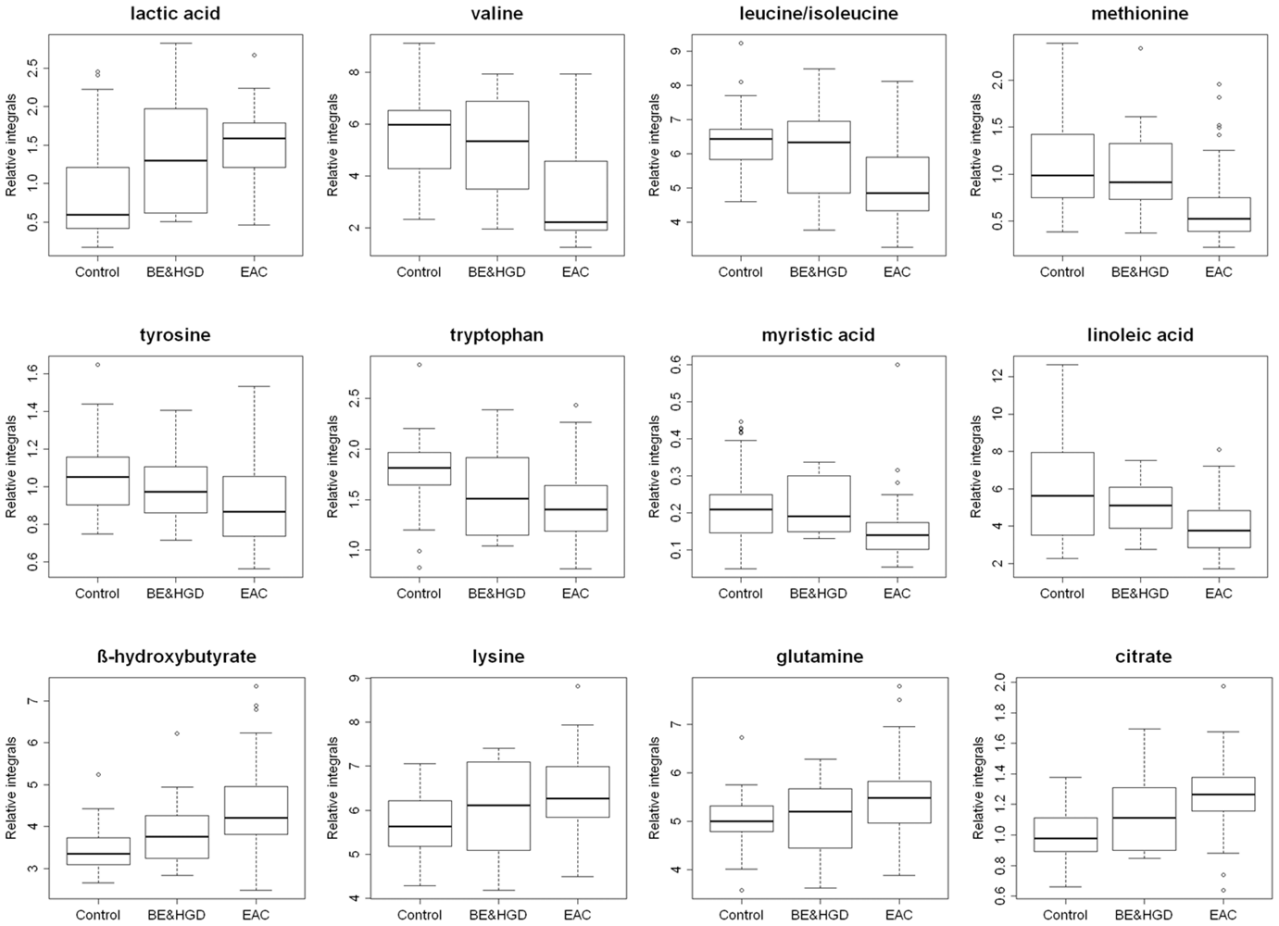
Box-and-whisker plots illustrating progressive changes of the metabolite levels in high-risk patients (BE and HGD) and esophageal adenocarcinoma (EAC) patients relative to healthy controls. Horizontal line in the middle portion of the box, median; bottom and top boundaries of boxes, lower and upper quartile; whiskers, 5th and 95th percentiles; open circles, outliers. The first eight markers were detected by LC-MS, and the remaining four were detected by NMR.

Levels of the metabolites between the three groups, EAC, BE & HGD, and healthy controls were compared using box-and-whisker plots. Interestingly, the average levels for 12 of the metabolites, including lactic acid, valine, leucine/isoleucine, methionine, tyrosine, tryptophan, myristic acid, linoleic acid, β-hydroxybutyrate, lysine, glutamine and citrate progressively changed with the average levels for BE and HGD patients falling in between the levels for healthy controls and EAC (Figure 3). While the levels for lactic acid, 3-hydroxybutyrate, lysine, glutamine and citrate increased, the levels for valine, leucine/isoleucine, methionine, tyrosine, tryptophan, myristic acid and linoleic acid decreased progressively.

**Figure 4:**
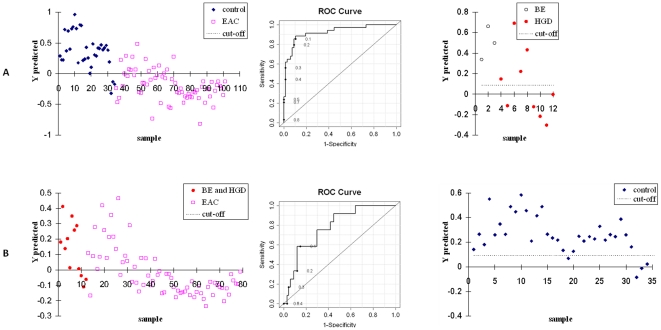
PLS-DA models comparing two patient groups, their coresponding ROC curves, and the prediction of the models for the other (third) patient group using the 12 trending markers of **Figure 3**. (A) Performance comparison of metabolic profiles between EAC patients and healthy controls, AUROC = 0.92. (B) Performance comparison of metabolic profiles between EAC and BE/HGD patients, AUROC = 0.78.

Using these 12 markers, PLS-DA models were again built using LC-MS and NMR separately and in combination, to test the classification accuracy for each of the two group comparisons (Figure 4). Figure 4A shows the PLS-DA model for EAC v. the healthy controls and predicts values for the high-risk patients. The model provided a sensitivity and specificity of 89% and 90%, respectively, with an AUROC of 0.92, although the predictive test for BE and HGD did not improve over that using the previous PLS-DA model (Figure 1B and C). Figure 4B shows the PLS-DA model comparing EAC and the high-risk patient group, resulting in a sensitivity, specificity and AUROC of 76% 70% and 0.78, respectively. In this case an improvement in the predictive testing of the control subjects was obtained, with 30 out of 34 controls appearing above the cut-off line (non-EAC like). However, these 12 markers could not be used to generate a clear classification between healthy controls and at risk patients using PLS-DA, therefore it is not possible to use such a model to predict EAC (data not shown).

## Discussion

This study is focused on identifying distinguishing metabolites for the establishment of improved clinical biomarkers for EAC detection, the development of a more robust classification model, and insights into the altered metabolic pathways in EAC.

The differentiating metabolites derived from the individual LC-MS and NMR analyses showed distinct differences in a number of metabolites between EAC and controls and achieved good classification accuracy. However, the combination of metabolic profiles from the two methods enabled access to an increased number of distinguishing metabolites. The predictive power of the model derived from the combination of MS and NMR methods performed better in both sensitivity and specificity when compared with the results from the individual analytical methods. The complementary nature of the combined metabolic pool derived from the two methods contributed to this improvement of the model. In fact, all of the LC-MS detected metabolite marker candidates except lactic acid are different from those detected by NMR. It should, however, be stressed that while the improvement in classification was very clear for distinguishing EAC and controls, when the two analytical methods were combined the improved performance for discriminating high risk (BE and HGD) patients versus EAC was less noticeable and only in the high specificity region (Figures 1 and 2). This effect is likely due to the small number of high risk patients and possibly to variation of metabolic alterations to a greater degree from one patient to another, both of which can make model prediction more challenging.

Comparison of the individual metabolites and the statistical models developed using the differentiating metabolites in the three groups showed that metabolic profiles of the BE and HGD patients were different from both EAC patients and healthy controls. Progressive changes in the levels of 12 metabolites derived from LC-MS and NMR methods indicate the potential utility for identifying BE and HGD patients who may develop EAC (Figure 3). This is particularly important since BE and HGD are major risk factors for the development of EAC. Identification of metabolites in these patients, which are potentially predictive of the development of EAC is particularly important for the management of at risk patients.

**Figure 5:**
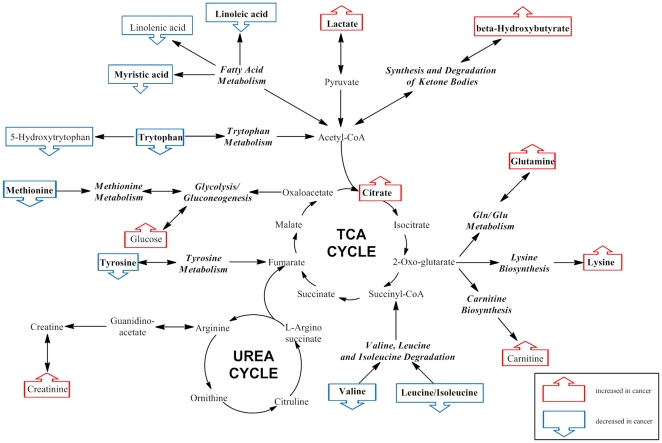
Altered metabolism pathways for the most relevant metabolic differences between patients with EAC and control subjects. Blue boxes indicate metabolites that are up-regulated in EAC patient sera, while red boxes indicate metabolites that are down-regulated. Metabolites in bold showed mean levels that changed progressively from control to high-risk esophagus diseases (BE and HGD) and ultimately EAC.

Identification of the metabolic pathways associated with specific metabolites displaying altered levels can improve the understanding of the biology and pathology in the trajectory from normal to esophageal disease and ultimately cancer. Previously, we showed a simplified pathway map based on the metabolite markers identified by NMR and compared the results with other type of cancers [27]. Building upon this model, Figure 5 shows a more detailed pathway map associated with metabolite markers identified using both MS and NMR methods. Altered pathways include changes in amino acid metabolism, biosynthesis and degradation (glutamine, lysine, carnitine, valine, leucine/isoleucine, methionine, tryptophan, 5-hydroxytrytophan, and tyrosine), glycolysis (lactate and glucose), ketone bodies synthesis and degradation (β-hydroxybutyate), tricarboxylic acid (TCA) cycle (citrate) and fatty acid metabolism (linoleic acid, linolenic acid and myristic acid).

Energy metabolism and the TCA cycle dominate the altered biochemistry of EAC. Accumulation of lactate and glucose, which is common in many cancers mirrors the demand for higher energy in tumor malignancy [32]. The increase of carnitine in the EAC patients indicates increased activity of carnitine, lysine and glutamine biosynthesis connected with the TCA cycle via lactate accumulation, again in response to the higher energy demand of the tumor. Many serum amino acids, including valine, leucine/isoleucine, tyrosine, methionine, tryptophan and 5-hydroxytrytophan, were down-regulated in EAC patients compared with healthy controls, which indicates an increased demand for and overutilization of amino acids in the tumor tissue, as further evidenced by other reports on the cancer [25], [26] as well as other malignant tumors [33], [34]. Fatty acid metabolism is also altered in the cancer patient sera, as seen by the reduced levels of a number of fatty acids, and which is also in accordance with findings in serum from other cancers such as colorectal cancer [35].

We also noticed that valine and tyrosine were decreased in the sera of patients in the current study, but increased in the tissue of EAC patients [25]. The intriguing differential regulation of certain metabolites in biofluids versus tissue samples for the same disease has been reported in other disease metabolic profiling studies as well. For example, while histidine increased in colorectal cancer patient tissue [36], it was depleted in urine [37]. Thus, to examine the intimate correlation of serum, urine and tissue metabolism as a whole, similar methodologies should be utilized to identify the metabolic alterations and pathways of the same study subjects in a broader range of sample types [38]. It would also be of interest to provide a more dynamic metabolic picture by determining metabolic fluxes or other changes in the metabolic pathways that may be altered in the presence of the disease [39], [40], [41].

In conclusion, we have shown that the metabolic profiling of serum using a combination of LC-MS and ^1^H NMR, along with multivariate statistical methods allows a detailed picture of metabolic changes in EAC and patients with high cancer risk (BE and HGD), compared with healthy controls. These patient groups can be distinguished from one another with good accuracy. Performance of the combination the two analytical methods is particularly striking for distinguishing EAC and controls. As the two analytical largely detect different metabolites, their combined use for global metabolic profiling is advantageous. However, the improved performance for discriminating high risk (BE and HGD) patients versus EAC is not large, and only noticeable for the high specificity region of the ROC curve. This result is likely due to the small number of patients and the relatively poor performance of the NMR detected markers in the challenging task of distinguishing at risk patient from those with EAC. Progressive changes in a number of metabolites between the three groups are particularly noteworthy since these metabolites, which vary gradually from controls to BE and HGD and EAC, may be potentially useful biomarkers to detect esophageal cancer early.

## Supporting Information

Figure S1 **Box-and-whisker plots illustrating differences between EAC patients, high-risk patients (BE and HGD) and healthy controls for the 12 markers detected from LC-MS.** Y-axis of each plot indicates the signal intensities.
(TIF)

Figure S2 **Comparison results for metabolic profiles from healthy controls with high-risk, BE and HGD, patients.** (A) Left, result of the PLS-DA model for the one metabolite from LC-MS; middle, ROC curve for the cross-validated predicted class values (AUROC = 0.76); right, PLS-DA prediction for the EAC samples using the same metabolite and cutoff. (B) Left, result of the PLS-DA model comparing healthy normals and high risk patients (BE & HDG) for the 4 markers detected by NMR; middle, ROC curve for the cross-validated predicted class values (AUROC = 0.80); right, PLS-DA prediction for the EAC samples using the model developed using NMR markers for high-risk indivduals and healthy controls. (C) Same as (B) except using the combination of 5 markers from LC-MS and NMR (AUROC = 0.80).
(TIF)

Table S1 **Demographic and clincial parameters for esophageal adenocarcinoma (EAC) patients.**
(DOCX)

Table S2 **Identification information for LC-MS detected metabolites.**
(DOCX)

Table S3 **Identification information for NMR detected metabolites.**
(DOCX)

Table S4 **Results of ANOVA for the different metabolite biomarker candidates detected by LC-MS and NMR.**
(DOCX)

Table S5 **Results of the t-test (** ***p*** **values) for comparing age, gender and cancer stage, separately, for esophageal cancer patients.**
(DOCX)
