# Supplementary figures and images for: Esophageal Cancer Metabolite Biomarkers Detected by LC-MS and NMR Methods
Source: PLoS One. 2012 Jan 23;7(1):e30181. doi: 10.1371/journal.pone.0030181 (PMC3264576; doi:10.1371/journal.pone.0030181)

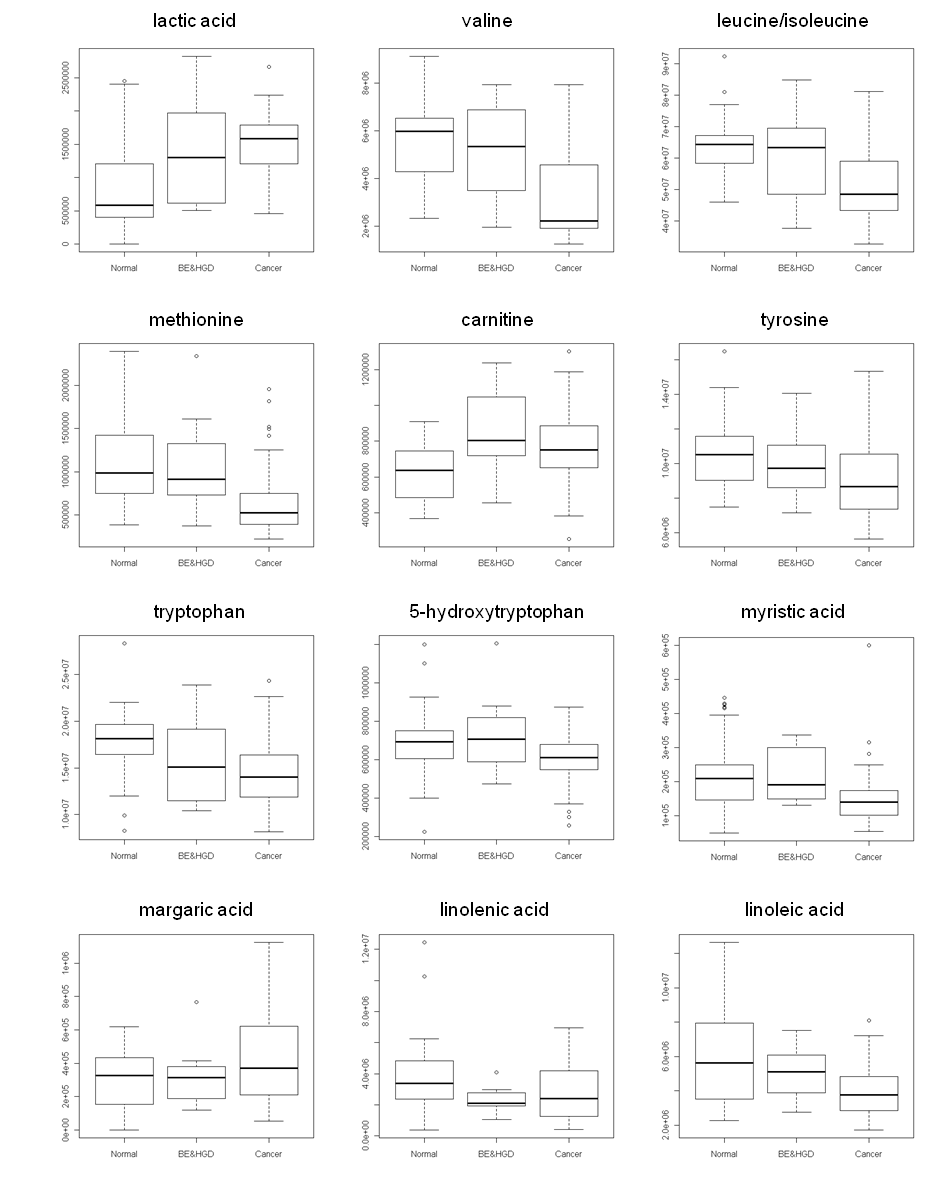

Supplement: Figure S1 — Box-and-whisker plots illustrating differences between EAC patients, high-risk patients (BE and HGD) and healthy controls for the 12 markers detected from LC-MS. Y-axis of each plot indicates the signal intensities. (TIF) [file pone.0030181.s001.tif]

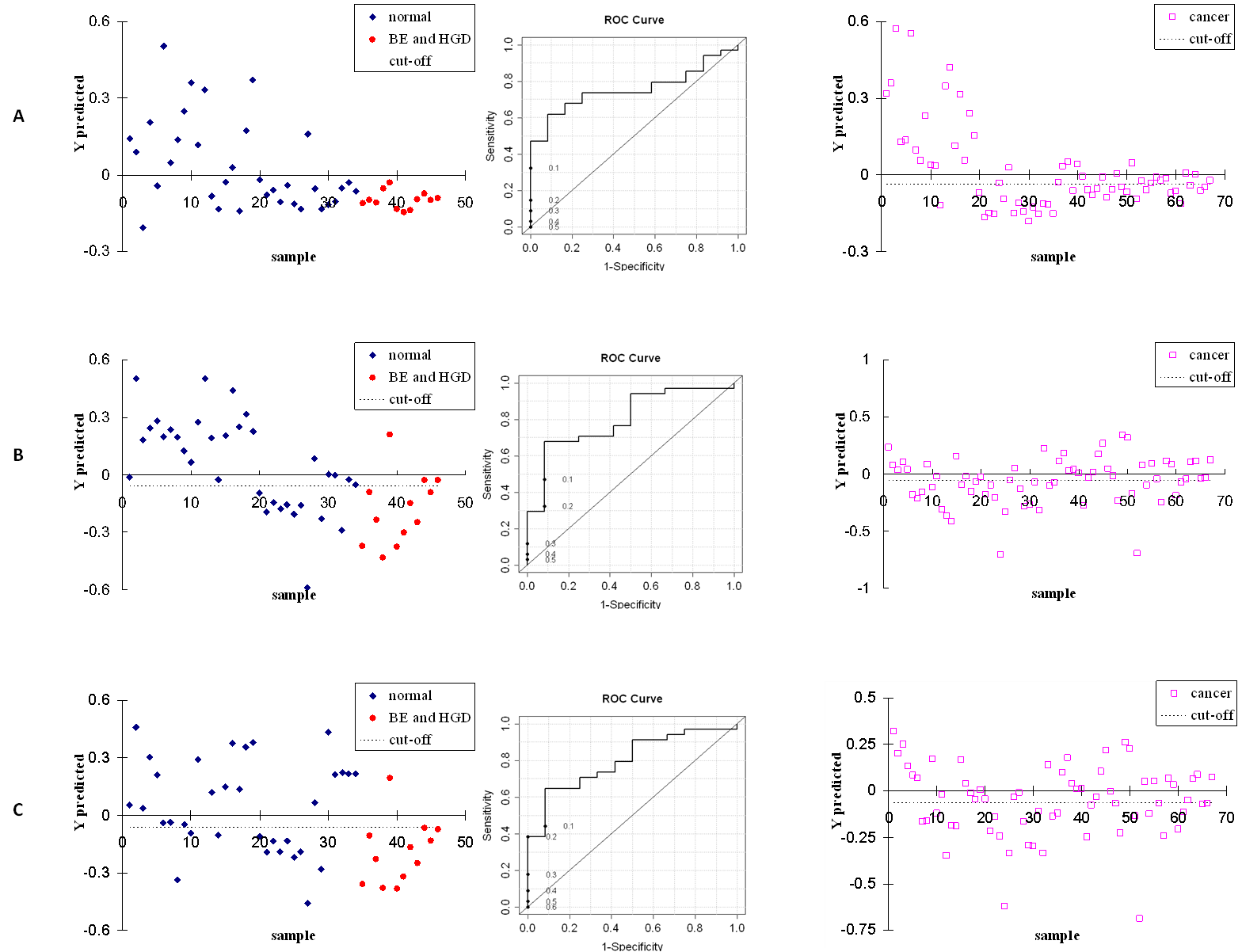

Supplement: Figure S2 — Comparison results for metabolic profiles from healthy controls with high-risk, BE and HGD, patients. (A) Left, result of the PLS-DA model for the one metabolite from LC-MS; middle, ROC curve for the cross-validated predicted class values (AUROC = 0.76); right, PLS-DA prediction for the EAC samples using the same metabolite and cutoff. (B) Left, result of the PLS-DA model comparing healthy normals and high risk patients (BE & HDG) for the 4 markers detected by NMR; middle, ROC curve for the cross-validated predicted class values (AUROC = 0.80); right, PLS-DA prediction for the EAC samples using the model developed using NMR markers for high-risk indivduals and healthy controls. (C) Same as (B) except using the combination of 5 markers from LC-MS and NMR (AUROC = 0.80). (TIF) [file pone.0030181.s002.tif]
