# Supplementary material for: Esophageal Cancer Metabolite Biomarkers Detected by LC-MS and NMR Methods
Source: PLoS One. 2012 Jan 23;7(1):e30181. doi: 10.1371/journal.pone.0030181 (PMC3264576; doi:10.1371/journal.pone.0030181)
Supplement: Table S1 — Demographic and clincial parameters for esophageal adenocarcinoma (EAC) patients. (DOCX) [file pone.0030181.s003.docx]

**Table S1:** Demographic and clincial parameters for esophageal adenocarcinoma (EAC) patients.

| **Parameter** | | **EAC patients (n=67)** |
| --- | --- | --- |
| Average age (range) | | 65.6 (46-91) |
| Number of male | | 56 |
| Cancer stage | T1 | 7 |
|  | T2 | 11 |
|  | T3 | 36 |
|  | T4 | 2 |
|  | N0 | 16 |
|  | N1 | 37 |
|  | M | 18 |
