# Supplementary material for: Esophageal Cancer Metabolite Biomarkers Detected by LC-MS and NMR Methods
Source: PLoS One. 2012 Jan 23;7(1):e30181. doi: 10.1371/journal.pone.0030181 (PMC3264576; doi:10.1371/journal.pone.0030181)
Supplement: Table S2 — Identification information for LC-MS detected metabolites. (DOCX) [file pone.0030181.s004.docx]

**Table S2:** Identification information for LC-MS detected metabolites.

| **Compound Name** | **Formula** | **m/z Calculated (Da)** | **m/z Detected (Da)** | **Delta**  **m/z (ppm)** | **RT Authentic Pooled (min)** | **RT Detected (min)** | **Delta RT (min)** |
| --- | --- | --- | --- | --- | --- | --- | --- |
| lactic acid | C3H6O3 | 90.0317 | 90.0318 | -1.18 | 0.51 | 0.49 | 0.02 |
| valine | C5H11NO2 | 117.0790 | 117.0790 | 0 | 0.38 | 0.42 | -0.04 |
| pyroglutamic acid | C5H7NO3 | 129.0426 | 129.0429 | -2.40 | 0.56 | 0.55 | 0.01 |
| leucine/isoleucine***^a^*** | C6H13NO2 | 131.0946 | 131.0944 | 1.75 | 0.59 | 0.62 | -0.03 |
| methionine | C5H11NO2S | 149.0511 | 149.0514 | -2.35 | 0.41 | 0.47 | -0.06 |
| carnitine | C7H15NO3 | 161.1052 | 161.1049 | 1.80 | 0.33 | 0.37 | -0.04 |
| tyrosine | C9H11NO3 | 181.0739 | 181.0734 | 2.71 | 0.49 | 0.51 | -0.02 |
| tryptophan | C11H12N2O2 | 204.0899 | 204.0898 | 0.39 | 1.38 | 1.43 | -0.05 |
| 5-hydroxytryptophan | C11H12N2O3 | 220.0848 | 220.0852 | -1.86 | 0.64 | 0.71 | -0.07 |
| myristic acid | C14H28O2 | 228.2089 | 228.2092 | -1.18 | 12.34 | 12.36 | -0.02 |
| margaric acid | C17H34O2 | 270.2559 | 270.2562 | -1.18 | 12.93 | 12.95 | -0.02 |
| linolenic acid | C18H30O2 | 278.2246 | 278.2252 | -2.23 | 11.29 | 11.33 | -0.04 |
| linoleic acid | C18H32O2 | 280.2402 | 280.2405 | -0.96 | 12.71 | 12.75 | -0.04 |

***^a^*** The structural isomers could not be separated with the current LC method.
