# Supplementary material for: Esophageal Cancer Metabolite Biomarkers Detected by LC-MS and NMR Methods
Source: PLoS One. 2012 Jan 23;7(1):e30181. doi: 10.1371/journal.pone.0030181 (PMC3264576; doi:10.1371/journal.pone.0030181)
Supplement: Table S3 — Identification information for NMR detected metabolites. (DOCX) [file pone.0030181.s005.docx]

**Table S3:** Identification information for NMR detected metabolites.

| **chemical shift (ppm)** ***^a^*** | **multiplicity*^b^*** | **assignment** |
| --- | --- | --- |
| 1.48 | d | alanine |
| 2.02 | s | N-acetylated protein |
| 2.09 | m | glutamine |
| 2.39 | m | β-hydroxybutyrate |
| 2.44 | m | glutamate |
| 2.53 | d | citrate |
| 2.63 | m | unknown1***^c^*** |
| 2.91 | m | unknown2***^d^*** |
| 3.00 | t | lysine |
| 3.35 | m | proline |
| 4.05 | s | creatinine |
| 4.11 | q | lactate |
| 5.22 | d | glucose |
| 7.03 | s | histidine |
| 7.18 | d | tyrosine |

*^a,b^*The chemical shift and multiplicity are NMR dependent quantities that indicate the spectral peak position and number of peaks, respectively, and allow the spectroscopist to identify the chemical compound (s=singlet; d=doublet; t=triplet; q=quartet; m=complex multiplet). *^c^*Unknown 1 was discovered by comparing the control group with EAC patients as reported in our previous work. *^d^*Unknown 2 was discovered by comparing the normal subjects with BE and HGD patients.
