# Supplementary material for: Esophageal Cancer Metabolite Biomarkers Detected by LC-MS and NMR Methods
Source: PLoS One. 2012 Jan 23;7(1):e30181. doi: 10.1371/journal.pone.0030181 (PMC3264576; doi:10.1371/journal.pone.0030181)
Supplement: Table S4 — Results of ANOVA for the different metabolite biomarker candidates detected by LC-MS and NMR. (DOCX) [file pone.0030181.s006.docx]

**Table S4:** Results of ANOVA for the different metabolite biomarker candidates detected by LC-MS and NMR.

| **Metabolite** | **Detection**  **Method** | ***p*-values from ANOVA** |
| --- | --- | --- |
| lactic acid | LC-MS | 2.38E-06 |
|  | NMR | 7.33E-06 |
| valine | LC-MS | 8.33E-07 |
|  | NMR | 2.48E-02 |
| leucine/isoleucine | LC-MS | 4.36E-06 |
| methionine | LC-MS | 2.93E-06 |
| carnitine | LC-MS | 2.49E-04 |
| tyrosine | LC-MS | 2.16E-02 |
|  | NMR | 1.31E-02 |
| tryptophan | LC-MS | 1.53E-04 |
| 5-hydroxytryptophan | LC-MS | 8.30E-03 |
| myristic acid | LC-MS | 4.36E-04 |
| margaric acid | LC-MS | 7.01E-02 |
| linolenic acid | LC-MS | 5.33E-03 |
| linoleic acid | LC-MS | 2.43E-06 |
| pyroglutamic acid | LC-MS | 4.44E-02 |
| glutamine | NMR | 3.76E-03 |
| β-hydroxybutyrate | NMR | 3.83E-06 |
| citrate | NMR | 4.01E-07 |
| unknown 1 | NMR | 1.48E-08 |
| lysine | NMR | 4.79E-04 |
| creatinine | NMR | 2.37E-02 |
| glucose | NMR | 1.87E-03 |
| N-acetylated protein | NMR | 9.26E-03 |
| proline | NMR | 2.39E-03 |
| histidine | NMR | 1.95E-02 |
| alanine | NMR | 3.22E-02 |
| glutamate | NMR | 7.39E-02 |
| unknown 2 | NMR | 2.04E-02 |
