# Supplementary material for: Esophageal Cancer Metabolite Biomarkers Detected by LC-MS and NMR Methods
Source: PLoS One. 2012 Jan 23;7(1):e30181. doi: 10.1371/journal.pone.0030181 (PMC3264576; doi:10.1371/journal.pone.0030181)
Supplement: Table S5 — Results of the t-test ( p values) for comparing age, gender and cancer stage, separately, for esophageal cancer patients. (DOCX) [file pone.0030181.s007.docx]

**Table S5:** Results of the t-test (*p* values) for comparing age, gender and cancer stage, separately, for esophageal cancer patients.

| **Metabolite** | **Detection** | **above-average age vs below-average age*^a^*** | **female vs male** | **T(any)N0 vs T(any)N1** | **T(any)N(any)M0 vs T(any)N(any)M1** |
| --- | --- | --- | --- | --- | --- |
| lactic acid | LC-MS | 0.65 | 0.70 | 0.99 | 0.36 |
|  | NMR | 0.84 | 0.70 | 0.82 | 0.10 |
| valine | LC-MS | 0.59 | 0.84 | 0.68 | 0.72 |
|  | NMR | 0.77 | 0.47 | 0.94 | 0.91 |
| leucine/isoleucine | LC-MS | 0.71 | 0.49 | 0.19 | 0.35 |
| methionine | LC-MS | 0.88 | 0.79 | 0.40 | 0.75 |
| carnitine | LC-MS | 0.07 | 0.07 | 0.15 | 0.95 |
| tyrosine | LC-MS | 0.26 | 0.25 | 0.39 | 0.56 |
|  | NMR | 0.33 | 0.65 | 0.76 | 0.99 |
| tryptophan | LC-MS | 0.12 | 0.56 | 0.68 | 0.26 |
| 5-hydroxytryptophan | LC-MS | 0.65 | 0.62 | 0.22 | 0.78 |
| myristic acid | LC-MS | 0.82 | 0.44 | 0.36 | 0.67 |
| margaric acid | LC-MS | 0.80 | 0.30 | 0.10 | 0.73 |
| linolenic acid | LC-MS | 0.95 | 0.13 | 0.10 | 0.45 |
| linoleic acid | LC-MS | 0.24 | 0.74 | 0.30 | 0.80 |
| pyroglutamic acid | LC-MS | 0.14 | 0.15 | 0.77 | 0.10 |
| glutamine | NMR | 0.96 | 0.53 | 0.13 | 0.34 |
| β-hydroxybutyrate | NMR | 0.73 | 0.60 | 0.52 | 0.52 |
| citrate | NMR | 0.10 | 0.31 | 0.83 | 0.47 |
| unknown 1 | NMR | 0.85 | 0.17 | 0.52 | 0.63 |
| lysine | NMR | 0.97 | 0.50 | 0.29 | 0.28 |
| creatinine | NMR | 0.07 | 0.14 | 0.32 | 0.26 |
| glucose | NMR | 0.32 | 0.74 | 0.40 | 0.62 |
| N-acetylated protein | NMR | 0.73 | 0.90 | 0.27 | 0.76 |
| proline | NMR | 0.52 | 0.10 | 0.20 | 0.70 |
| histidine | NMR | 0.28 | 0.93 | 0.38 | 0.25 |
| alanine | NMR | 0.50 | 0.84 | 0.94 | 0.67 |
| glutamate | NMR | 0.40 | 0.29 | 0.74 | 0.44 |
| unknown 2 | NMR | 0.30 | 0.09 | 0.78 | 0.66 |

*^a^*average age of all cancer patients = 65.7 yrs
